# Single mutation makes *Escherichia coli* an insect mutualist

**DOI:** 10.1038/s41564-022-01179-9

**Published:** 2022-08-04

**Authors:** Ryuichi Koga, Minoru Moriyama, Naoko Onodera-Tanifuji, Yoshiko Ishii, Hiroki Takai, Masaki Mizutani, Kohei Oguchi, Reiko Okura, Shingo Suzuki, Yasuhiro Gotoh, Tetsuya Hayashi, Masahide Seki, Yutaka Suzuki, Yudai Nishide, Takahiro Hosokawa, Yuichi Wakamoto, Chikara Furusawa, Takema Fukatsu

**Affiliations:** 1 Bioproduction Research Institute, National Institute of Advanced Industrial Science and Technology, Tsukuba, Japan; 2 Department of Basic Science, Graduate School of Arts and Sciences, The University of Tokyo, Tokyo, Japan; 3 Center for Biosystem Dynamics Research, RIKEN, Osaka, Japan; 4 Department of Bacteriology, Faculty of Medical Sciences, Kyushu University, Fukuoka, Japan; 5 Laboratory of Systems Genomics, Department of Computational Biology and Medical Sciences, Graduate School of Frontier Sciences, The University of Tokyo, Chiba, Japan; 6 National Agriculture and Food Research Organization, Institute of Agrobiological Sciences, Tsukuba, Japan; 7 Department of Biology, Faculty of Science, Kyushu University, Fukuoka, Japan; 8 Universal Biology Institute, The University of Tokyo, Tokyo, Japan; 9 Department of Biological Sciences, The University of Tokyo, Tokyo, Japan; 10 Graduate School of Life and Environmental Sciences, University of Tsukuba, Tsukuba, Japan

**Keywords:** Experimental evolution, Symbiosis, Bacterial evolution

## Abstract

Microorganisms often live in symbiosis with their hosts, and some are considered mutualists, where all species involved benefit from the interaction. How free-living microorganisms have evolved to become mutualists is unclear. Here we report an experimental system in which non-symbiotic *Escherichia coli* evolves into an insect mutualist. The stinkbug *Plautia stali* is typically associated with its essential gut symbiont, *Pantoea* sp., which colonizes a specialized symbiotic organ. When sterilized newborn nymphs were infected with *E. coli* rather than *Pantoea* sp., only a few insects survived, in which *E. coli* exhibited specific localization to the symbiotic organ and vertical transmission to the offspring. Through transgenerational maintenance with *P. stali*, several hypermutating *E. coli* lines independently evolved to support the host’s high adult emergence and improved body colour; these were called ‘mutualistic’ *E. coli*. These mutants exhibited slower bacterial growth, smaller size, loss of flagellar motility and lack of an extracellular matrix. Transcriptomic and genomic analyses of ‘mutualistic’ *E. coli* lines revealed independent mutations that disrupted the carbon catabolite repression global transcriptional regulator system. Each mutation reproduced the mutualistic phenotypes when introduced into wild-type *E. coli*, confirming that single carbon catabolite repression mutations can make *E. coli* an insect mutualist. These findings provide an experimental system for future work on host–microbe symbioses and may explain why microbial mutualisms are omnipresent in nature.

## Abstract (web summary)

Experimental evolution of *Escherichia coli* in an insect host reveals that a single mutation in the carbon catabolite repression pathway can transform a non-symbiotic microbial species into an insect mutualist.

## Main

Microbial symbioses are among the major evolutionary drivers underpinning biodiversity, wherein relationships range from parasitism through commensalism to mutualism^1,2^. Originally, however, such microbial symbionts must have been without association with their host organisms, deriving from environmental microbes at the beginning. How ordinary free-living microbes have become sophisticated mutualists is an important but unanswered question. To address this fundamental issue, experimental evolutionary approaches may provide valuable insights^3–9^. If a model microbe like *Escherichia coli* with elaborate molecular genetic tools and resources can establish a mutualistic association with a host organism via experimental evolution, such a ‘model experimental symbiotic system’ will be extremely useful for understanding the evolutionary processes of symbiosis towards mutualism. Recently, the stinkbug *Plautia stali* (Hemiptera: Pentatomidae) has emerged as an experimentally tractable model system for investigating the diversity, evolution and mechanism of gut symbiosis with bacterial mutualists^10,11^. In this study, we report an experimental system in which *E. coli* evolves into a bacterial mutualist that supports survival and reproduction of *P. stali*, thereby demonstrating that evolution of mutualism can proceed very easily and quickly via disruption of a global transcriptional regulator system.

## Results

### *E. coli* is potentially capable of symbiosis with *P. stali*

**Fig. 1:**
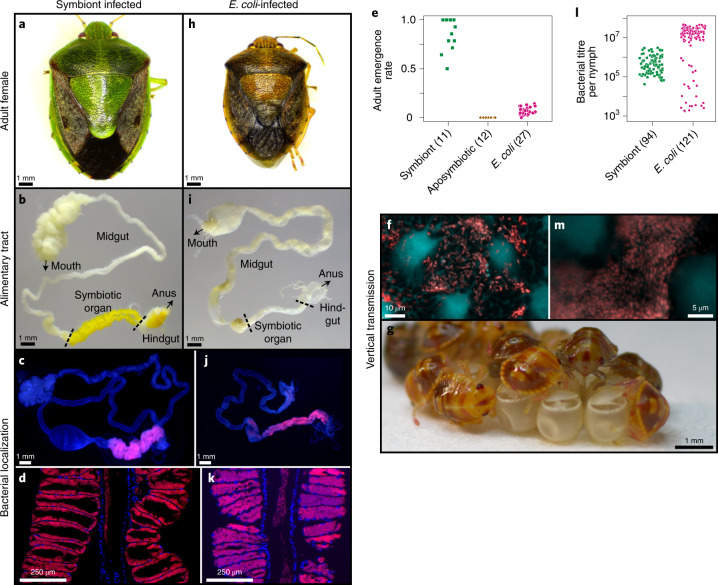
Infection, localization and vertical transmission of *E. coli* in the gut symbiotic system of *P. stali*. **a**, Normal symbiotic adult female, large in size and green in colour. **b**, Dissected alimentary tract, in which the symbiotic organ is well developed and yellow in colour. **c**, FISH localization of symbiont cells to the symbiotic organ. **d**, Magnified FISH image showing symbiont localization to crypt cavities of the symbiotic organ. The image is reconstructed by merging three microscopic images. **e**, Adult emergence rates of newborn nymphs inoculated with normal symbiont (*Pantoea* sp. A), no bacteria (aposymbiotic) and *E. coli*. **f**, Symbiont cells smeared on egg surface. **g**, Newborn nymphs sucking symbiont cells from the eggshell (Supplementary Video 1). **h**, *E. coli*-infected adult female, dwarf in size and brown in colour. **i**, Dissected alimentary tract, in which the symbiotic organ is atrophied. **j**, FISH localization of *E. coli* to the symbiotic organ. **k**, Magnified FISH image visualizing *E. coli* localization to crypt cavities of the symbiotic organ. **l**, Bacterial titres in symbiont-inoculated and *E. coli*-inoculated nymphs 1 d after second instar moult in terms of *groEL* and *nptII* gene copies per insect, respectively. **m**, *E. coli* cells smeared on the egg surface. **e**,**l**, The numbers of biological replicates are shown after the strain names. Level adjustments without non-linear change were applied to **c**, **d**, **f**, **j**, **k** and **m**. Source data

**Extended Data Fig. 1:**
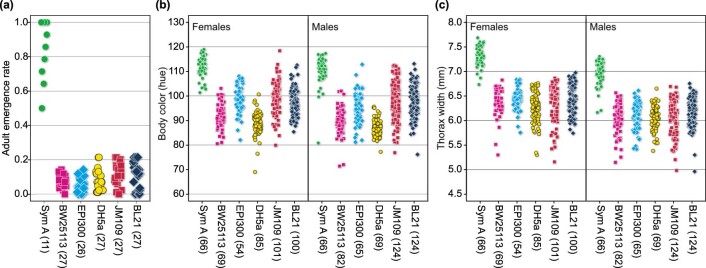
Phenotypes of *P. stali* adults infected with laboratory strains of *E. coli*. **a**, Adult emergence rate. **b**, Body colour (greenish hue) of females (left) and males (right). (**c**) Body size (thorax width) of females (left) and males (right). Sym A is *Pantoea* sp. A, the original, uncultivable and essential gut symbiont of *P. stali*^10^. BW25113, EPI300, DH5a, JM109 and BL21 are commonly used laboratory strains of *E. coli*. The numbers of biological replicates are shown after the strain names. Source data

**Extended Data Fig. 2:**
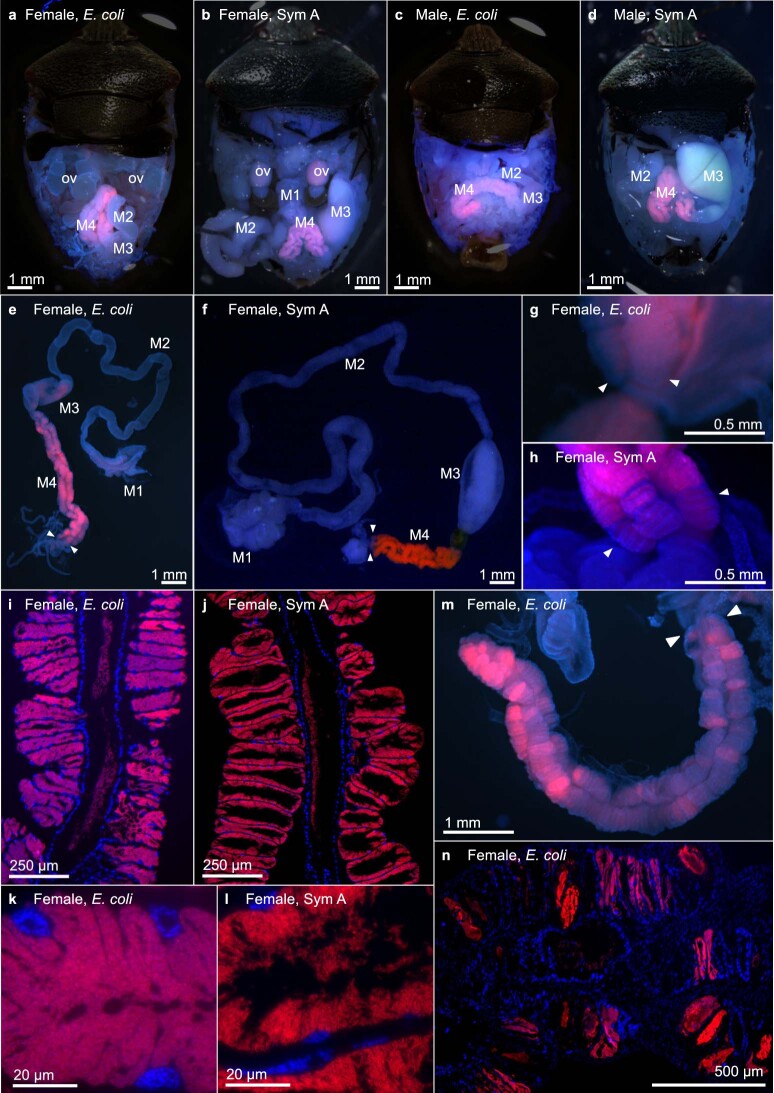
FISH localization of *E. coli* and original symbiont *Pantoea* sp. A (=Sym A) in *P. stali*. **a**–**d**, Localization in abdominal body cavity of adult insects: (**a**) *E. coli* in adult female, (**b**) Sym A in adult female, (**c**) *E. coli* in adult male, and (**d**) Sym A in adult male. FISH signals are localized to the midgut M4 region. Signals in oocytes are due to autofluorescence. Abbreviations: M1, M2, M3, and M4, midgut regions M1, M2, M3, and M4 (=symbiotic organ); ov, ovary. **e**,**f**, Localization of *E. coli* (**e**) and Sym A (**f**) in dissected alimentary tract of adult females. Arrowheads indicate female-specific enlarged end crypts at the posterior end of the symbiotic organ, which are presumably involved in vertical symbiont transmission by storing bacteria-containing secretion^49^. **g**,**h**, Magnified images of the end crypts infected with *E. coli* (**g**) and Sym A (**h**). Note that *E. coli*-infected end crypts are atrophied in comparison with Sym A-infected ones. **i**,**j**, Localization of *E. coli* (**i**) and Sym A (**j**) in the crypt cavities of the symbiotic organ. **k**,**l**, Magnified images of *E. coli* cells (**k**) and Sym A cells (**l**) packed in the crypt cavity. **m**,**n**, Patchy localization patterns of *E. coli* in the symbiotic organ, which are often found with *E. coli* but seldom observed with Sym A. Red signals represent the distribution of bacteria, except for red autofluorescence of ovaries in (**a**) and (**b**). **j** is reconstructed by merging five microscopic images. The level adjustment without non-linear change is applied to the images.

Plant-sucking heteropteran bugs generally possess specific symbiotic bacteria in the midgut, which contribute to their growth and survival via provisioning of essential amino acids and/or vitamins^12,13^. The brown-winged green stinkbug *P. stali* (Hemiptera: Pentatomidae) (Fig. 1a) developed a specialized symbiotic organ consisting of numerous crypts in a posterior region of the midgut (Fig. 1b). The crypt cavities are densely populated by a specific bacterial symbiont of the genus *Pantoea* (Fig. 1c,d). The symbiont is essential for growth and survival of the host insect. Normal insects infected with the uncultivable obligatory symbiont, *Pantoea* sp. A^10,14^, attained over 70% adult emergence rates (Fig. 1e), smeared the symbiont cells onto the eggs on oviposition (Fig. 1f) and transmitted the symbiont vertically to the offspring via nymphal probing of the eggshell (Fig. 1g and Supplementary Video 1). Aposymbiotic insects generated by egg surface sterilization died out with no adult emergence (Fig. 1e). Non-symbiotic bacteria, such as *Bacillus subtilis* and *Burkholderia insecticola*, cannot establish infection and symbiosis with *P. stali*^10^. Meanwhile, when *E. coli* was inoculated to sterilized newborn nymphs, the insects certainly exhibited retarded growth and high mortality; however, a small number of adult insects emerged, attaining 5–10% adult emergence rates (Fig. 1e and Extended Data Fig. 1)^10^. Such adult insects, which were dwarf in size and dark in colour (Fig. 1h), tended to die early but some insects managed to survive, mate and produce a small number of eggs. We dissected and inspected these insects, and found that, surprisingly, although the symbiotic organ was atrophied (Fig. 1i), *E. coli* localized to the midgut crypts just like the original symbiont, although the infection patterns were often patchy (Fig. 1j,k and Extended Data Fig. 2). Furthermore, *E. coli* cells were smeared on the eggshell and vertically transmitted to the offspring (Fig. 1l,m), although the transmission rates and the infection titres were unstable in comparison with those of the original symbiont (Fig. 1l). These results suggested that, although incipiently, *E. coli* is capable of localized infection, vertical transmission and supporting host survival in *P. stali*. Considering that *E. coli* belongs to the same Enterobacteriaceae as the original *Pantoea* symbiont, *E. coli* may be able to co-opt the mechanisms for infection and localization of the symbiont to establish the incipient symbiosis^10^. In this context, it seems relevant that, in the stinkbug family Pentatomidae, gut symbiotic bacteria have evolved repeatedly from the Enterobacteriaceae through recurrent acquisitions and replacements^15,16^.

### Experimental evolution using hypermutating *E. coli*

**Fig. 2:**
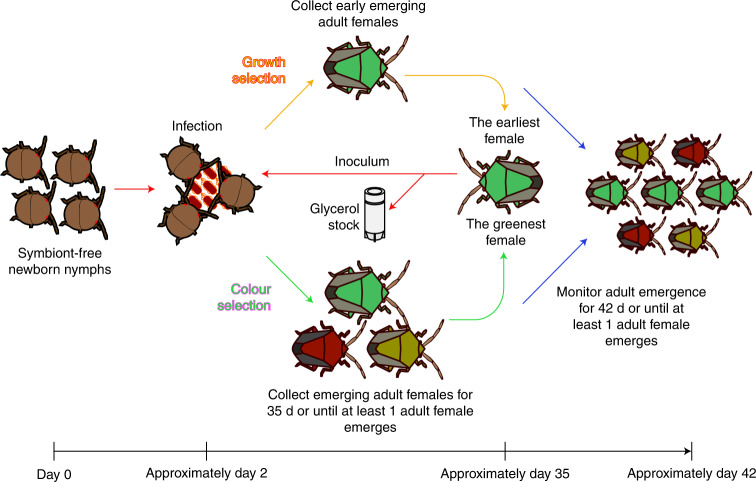
Experimental scheme for the evolution of mutualistic *E. coli* with *P. stali*. Evolutionary lines of *E. coli* were inoculated to sterilized newborn nymphs of *P. stali* and subjected to either host growth selection or host colour selection in this way.

This finding prompted us to apply experimental evolutionary approaches to the *P. stali*-*E. coli* relationship. By continuous inoculation to and maintenance with *P. stali*, would *E. coli* improve the symbiosis-related traits and finally evolve into a symbiont-like entity? Considering the expected difficulty in observing the evolution of elaborate symbiosis in a realistic time frame, we adopted the hypermutating the *E. coli* strain, Δ*mutS*, in which the DNA mismatch repair enzyme gene *mutS* is disrupted and the molecular evolutionary rate is elevated by two orders of magnitude^17^. The *E. coli* strain of the same genetic background, Δ*intS*, in which the phage integrase gene is disrupted without phenotypic consequences, was used as control. Two selection schemes, growth and colour selection, were conducted (Fig. 2). In growth selection lines (GmL for hypermutating Δ*mutS* lines; GiL for non-mutating Δ*intS* lines), the first-emerged adult insect was subjected to dissection of the symbiotic organ for inoculation to the next generation as well as freeze-storing (for example, GmL07G12 is **G**rowth-selected Δ***m****utS* **L**ine **07**, **G**eneration **12**). In colour selection lines (CmL for Δ*mutS* lines; CiL for Δ*intS* lines), the most greenish adult insect was subjected to dissection of the symbiotic organ for inoculation to the next generation as well as freeze storing (for example, CiL05G02 is **C**olour-selected Δ***i****ntS* **L**ine **05**, **G**eneration **02**). Throughout the evolutionary experiments, the host insects were supplied from a mass-reared inbred population of *P. stali*, thereby homogenizing the host genetic background and focusing on the evolutionary changes of the *E. coli* side. Since it takes around 1 month for newborn nymphs of *P. stali* to become adults under the rearing condition, it was expected that, ideally, we would be able to run 12 host generations per year. Actually, however, it took almost two years because (1) the *E. coli*-inoculated insects generally exhibited high mortality and retarded growth, (2) for keeping the insects under a good condition, frequent care without overcrowding was essential, which limited the manageable number of insects per evolutionary line ranging from 50 to 100 and (3) consequently, extended generation time and stochastic extinction of the evolutionary lines occurred frequently, which had to be restarted from the frozen *E. coli* stocks.

### Evolution of mutualistic *E. coli*

**Fig. 3:**
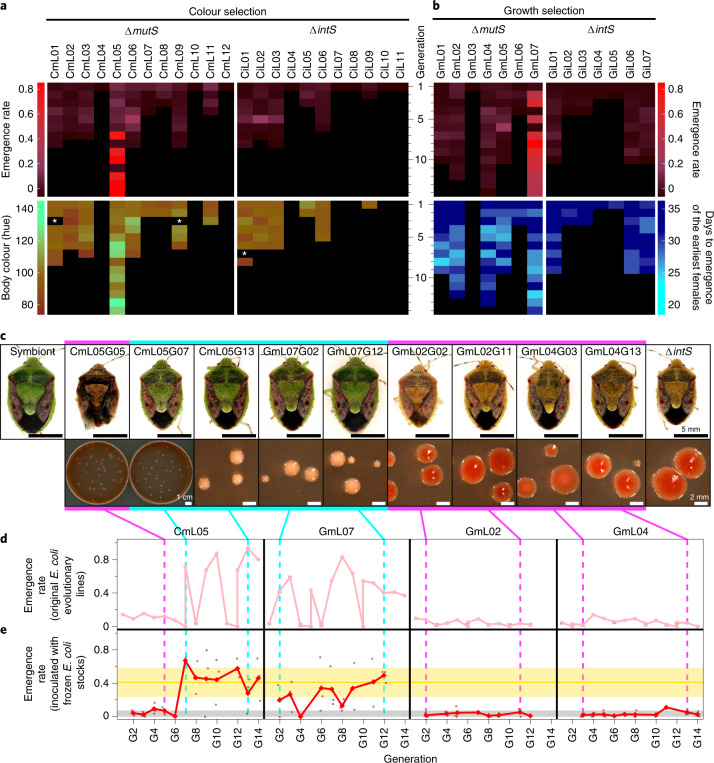
Evolution of mutualistic traits for *P. stali* in hypermutating *E. coli* lines. **a**, Evolutionary *E. coli* lines subjected to the host’s body colour selection. Data of adult emergence rate and body colour are displayed by heatmaps. The white asterisks indicate missing data of body colour measurement. **b**, Evolutionary *E. coli* lines subjected to the host’s growth speed selection. Data of adult emergence rate and days to the first adult emergence are displayed by heatmaps. **a**,**b**, When an evolutionary line produced no adult insect and recovery from the freeze stock failed twice consecutively, the evolutionary line was terminated due to shortage of inoculum. From generation 10 and onwards, selected evolutionary lines were maintained. **c**, Host’s body colour and colony morphology of evolutionary *E. coli* lines. Red colonies are due to rich extracellular matrix produced on the agar plates containing Congo red. **d**,**e**, Adult emergence patterns of *P. stali* infected with the representative *E. coli* lines, CmL05, GmL07, GmL02 and GmL04, in the original evolutionary experiments (**d**) and those in the confirmation experiments using frozen *E. coli* stocks (**e**). **d**, Pink lines represent the emergence rates of the original *E. coli* evolutionary lines, whereas the red lines in **e** represent the mean emergence rates (*n* = 3 biological replicates shown with brown dots) of the frozen *E. coli* stock infection experiments. **c**,**e**, The magenta and blue lines highlight ‘non-improved’ and ‘improved’ generations, respectively. Source data

**Extended Data Fig. 3:**
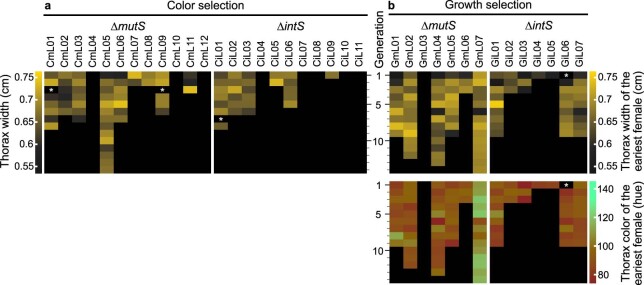
Effects of evolutionary *E. coli* lines on body size and colour of *P. stali*. **a**, Evolutionary *E. coli* lines subjected to host’s body colour selection. Data of host’s body width are displayed by heat maps. Also see Fig. 3a. **b**, Evolutionary *E. coli* lines subjected to host’s growth speed selection. Data of host’s body width and colour are displayed by heat maps. Also see Fig. 3b. Source data

**Extended Data Fig. 4:**
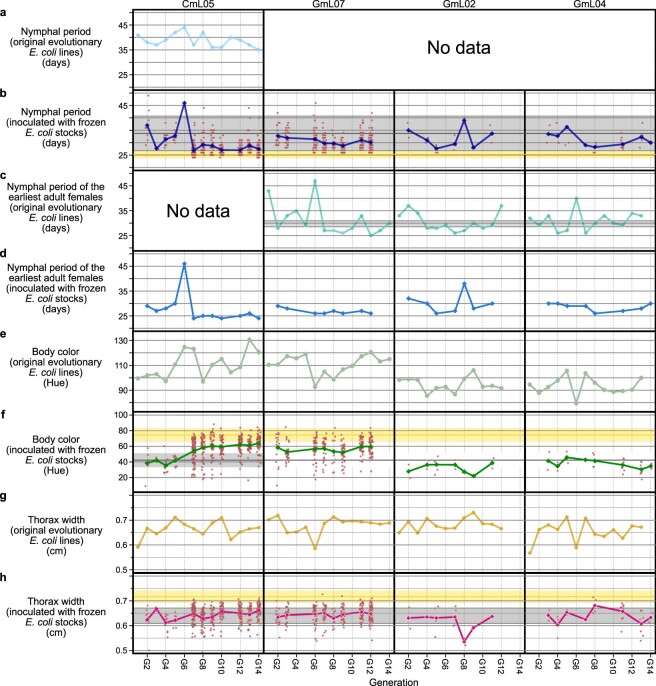
Adult phenotypes of *P. stali* infected with the evolutionary *E. coli* lines CmL05, GmL07, GmL02 and GmL04. **a**,**b**, Nymphal period. **c**,**d**, Nymphal period of the earliest adult females. **e**,**f**, Body colour. **g**,**h**, Thorax width. **a**,**c**,**e**,**g**, Phenotypes of the adult females used for inoculation to the next generation and preparation of glycerol stocks of the original evolutionary *E. coli* lines. **b**,**d**,**f**,**h**, Phenotypes of the adult insects inoculated with the frozen *E. coli* stocks. In **b**, **f** and **h**, line charts show mean values while dots indicate individual data points. Note that, corresponding to each original evolutionary *E. coli* line, three insect groups were inoculated with the frozen *E. coli* stock. In **b**, **f** and **h**, the yellow bands indicate the typical phenotypic ranges of the control insects infected with the original symbiont. Source data

We established and maintained 12 CmL colour selection lines with 11 CiL control lines and 7 GmL growth selection lines with 7 GiL control lines (Fig. 3a,b). While the control Δ*intS*-infected lines almost constantly exhibited low adult emergence rates, some of the hypermutating Δ*mutS*-infected lines started to produce more adult insects. Notably, in the colour selection line CmL05, the adult emergence rate jumped up at generation seven and high emergence rates were maintained thereafter (Fig. 3a). In the growth selection line GmL07, the adult emergence rate improved as early as at generation two, which was maintained thereafter (Fig. 3b). In CmL05 and GmL07, coincident with the improvement of the adult emergence rate, the body colour of the adult insects improved from dark to greenish (Fig. 3a–c and Extended Data Fig. 3); furthermore, the colony morphology of *E. coli* changed from large and flat with rich extracellular matrix to small and convex with little extracellular matrix (Fig. 3c). When the frozen stocks of CmL05 and GmL07 were inoculated to *P. stali*, the improved adult emergence rate, the greenish body colour and the small and convex colony shape were reproducibly observed (Fig. 3d,e and Extended Data Fig. 4). These results indicated that some evolutionary lines of hypermutating *E. coli* have evolved mutualistic traits for the host insect and that the phenotypic effects are attributable to genetic changes in the evolutionary *E. coli* lines.

### Microbial traits of mutualistic *E. coli*

**Fig. 4:**
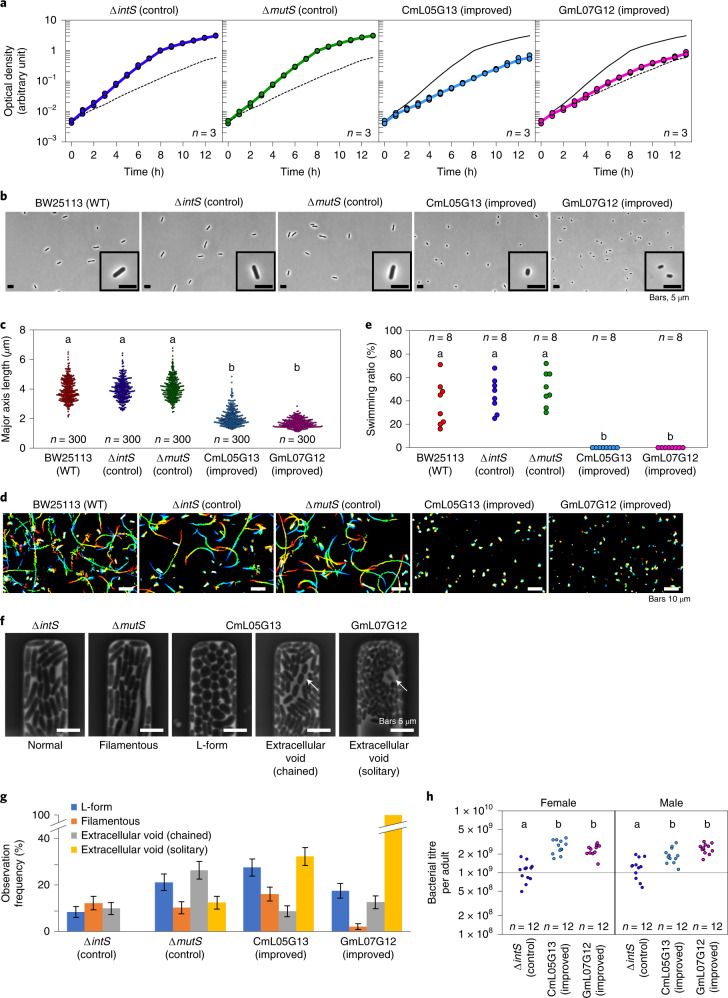
Microbial traits of evolutionary *E. coli* lines CmL05 and GmL07 in comparison with the original *E. coli* strains BW25113, Δ*intS* and Δ*mutS* cultured in liquid medium. **a**, Growth curves (three replicates each). The upper solid line is the trace of the Δ*intS* growth curve, whereas the lower dotted line is the trace of the CmL05 growth curve (Supplementary Video 2). **b**, Morphology of bacterial cells. **c**, Quantification of cell size in terms of major axis length. **d**, Motility of bacterial cells visualized by rainbow plot for 2 s (Supplementary Video 3). **e**, Quantification of bacterial motility in terms of the number of swimming cells per 100 cells observed. **f**, Characteristic cellular shape and growth mode in microfluidic channels. From left to right, the micrographs show the microchannels harbouring *E. coli* cells with normal rod-like shape (Δ*intS*), filamentation shape (Δ*mutS*), L form-like round shape (CmL05), extracellular void space and chained growth (CmL05) and extracellular void space and solitary growth (GmL07). The arrows indicate the cells showing the extracellular void space (Supplementary Video 4). **g**, Frequency of the microchannels in which *E. coli* cells exhibited characteristic cell shape and growth mode. The total numbers of microchannels observed in the time-lapse measurements (*N*) were 131 (Δ*intS*), 137 (Δ*mutS*), 149 (CmL05G13) and 143 (GmL07G12). The error bars represent the s.e. for the mean of binomially distributed samples, that is,$\sqrt {P\left( {1 - P} \right)/N}$, where *P* = *C*/*N* and *C* is the number of microchannels in which the cells with the indicated phenotype appeared. **h**, Bacterial titres in adult females 35 d after emergence in terms of *ntpII* gene copies per insect. **a**,**c**,**e**,**h**, The numbers of biological replicates are shown. **c**,**e**,**h**, Different alphabetical letters indicate statistically significant differences (two-sided pairwise Wilcoxon rank-sum test with Bonferroni correction: *P* < 0.05). The exact *P* values are provided with the source data. Source data

**Extended Data Fig. 5:**
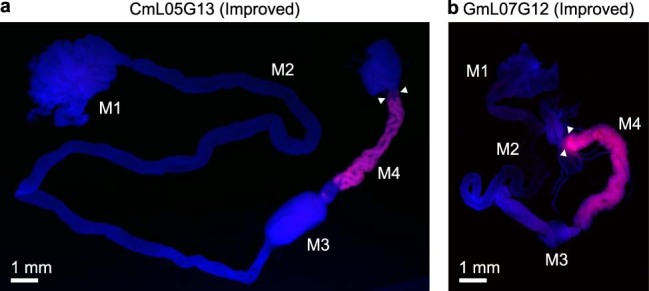
FISH localization of the improved evolutionary *E. coli* lines CmL05G13 and GmL07G12 in *P. stali*. **a**, CmL05G17. **b**, GmLG12. FISH signals are localized to the midgut M4 region. Abbreviations: M1, M2, M3, and M4, midgut regions M1, M2, M3, and M4 (=symbiotic organ). Arrowheads indicate female-specific enlarged end crypts at the posterior end of the symbiotic organ, which are presumably involved in vertical symbiont transmission by storing bacteria-containing secretion^49^. The level adjustment without non-linear change is applied to the images.

In addition to colony size, shape and extracellular matrix on agar plates (Fig. 3c), the mutualistic *E. coli* lines CmL05 and GmL07 in culture exhibited distinct microbial traits in comparison with the original *E. coli* strains: slower growth rate; smaller cell size; loss of flagellar motility; and unstable cell shape (Fig. 4a–g and Supplementary Videos 2–4). Within the host insect, the evolutionary *E. coli* lines CmL05 and GmL07 showed significantly higher infection densities than the original *E. coli* strains (Fig. 4h and Extended Data Fig. 5). These observations revealed that mutualistic *E. coli* lines certainly have evolved a variety of ‘symbiont-like’ microbial traits.

### Transcriptomics and genomics of mutualistic *E. coli*

**Fig. 5:**
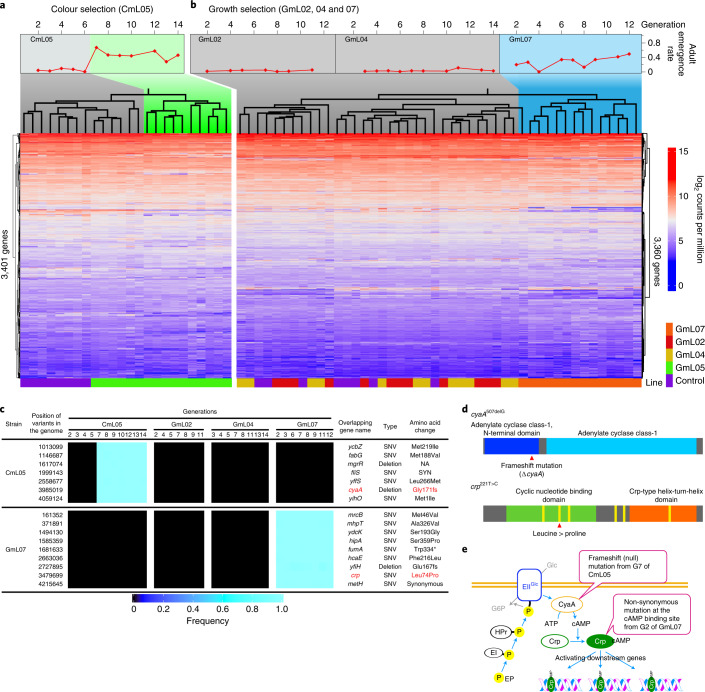
Transcriptomics and genomics of evolutionary *E. coli* lines. **a,b**, Clustering dendrograms and heatmaps based on gene expression levels across generations of evolutionary *E. coli* lines subjected to colour (left, 3,401 genes) (**a**) and growth selection (right, 3,360 genes) (**b**). The dendrograms represent the hierarchical clustering of the *E. coli* RNA-seq libraries. The grey and coloured areas depict non-improved and improved generations, respectively. **c**, Mutations identified in the genomes of CmL05 and GmL07 as coincident with the improvement of host phenotypes. SNV, single nucleotide variant. **d**, Candidate mutations disrupting the carbon catabolite repression: a frame shift mutation in *cyaA* of CmL05 (top) and a non-synonymous mutation causing change from leucine to proline at a functionally important cAMP binding domain in *crp* of GmL07 (bottom). **e**, Schematic presentation as to how the carbon catabolite repression pathway is disrupted by the *cyaA* and *crp* mutations. Source data

An aliquot of the dissected symbiotic organ from each generation of the colour selection line CmL05 was subjected to RNA sequencing, from which *E. coli*-derived reads were extracted and analysed (Supplementary Table 1). Interestingly, the gene expression patterns of *E. coli* at generations 7–14 after the improvement of host phenotypes were separately clustered in contrast to those at generations 1–6 before the improvement (Fig. 5a). In the growth selection line GmL07, similarly, the gene expression patterns of *E. coli* at generations 2–12 after the improvement were distinct from that at generation 1 before the improvement and also from those of the other growth selection lines GmL02 and GmL04 in which the improvement of host phenotypes did not occur (Fig. 5b). These results suggested that the evolution of the mutualistic *E. coli* lines entails a specific and global change of gene expression patterns.

**Extended Data Fig. 6:**
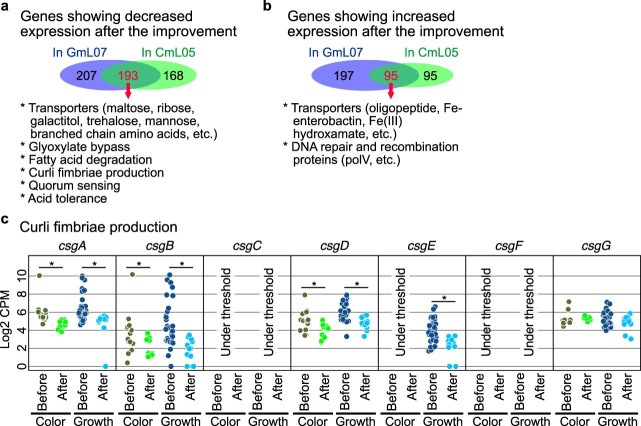
Gene expression changes of evolutionary *E. coli* lines GmL07 and CmL05 before and after improvement of host phenotypes. **a**,**b**, Venn diagrams showing down-regulated genes (**a**) and up-regulated genes (**b**) after the improvement of host phenotypes. **c**, Expression levels of genes involved in extracellular matrix (Curli fimbriae) production before and after the improvement of host phenotypes. Asterisks indicate statistically significant differences (FDR q-value < 0.01). The numbers of the biological replicates and exact FDR q-values are provided with the source data. Source data

In the growth selection line GmL07 and colour selection line CmL05, we surveyed differentially expressed genes before and after the improvement of host phenotypes, which identified 193 commonly downregulated genes and 95 commonly upregulated genes across GmL07 and CmL05 (Extended Data Fig. 6a,b). The commonly downregulated genes contained a number of metabolism-related genes, such as transporter genes for sugars and other nutrients like maltose, ribose, galactitol, trehalose, mannose and branched chain amino acids, glyoxylate bypass genes, fatty acid degradation genes and others. Notably, core genes involved in extracellular matrix (= Curli fimbriae) production were significantly downregulated after the improvement (Extended Data Fig. 6c), which likely accounted for the altered colony morphology of *E. coli* associated with the improvement of host phenotypes (Fig. 3c).

**Extended Data Fig. 7:**
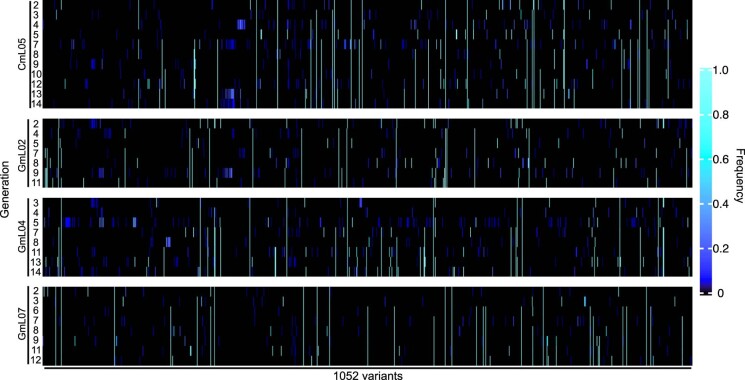
Mutations in the genomes of evolutionary *E. coli* lines CmL05, GmL02, GmL04 and GmL07 in the experimental evolutionary course. Frequencies of 1,052 variants identified in the experimental evolution lines and generations are colour-coded. Vertical axis represents the generations of the experimental evolution lines whereas horizontal axis represents an array of 1,052 variants. Source data

The improved lines CmL05 and GmL07 and the non-improved lines GmL02 and GmL04 were subjected to genome sequencing throughout the evolutionary course (Supplementary Table 2), which identified many mutations accumulated in the hypermutating *E. coli* lines (Extended Data Fig. 7 and Supplementary Table 3). In an attempt to identify candidate mutations that are correlated with the improvement of the host’s phenotype, we surveyed the mutations that appeared at generation seven of CmL05 and then fixed, which yielded seven candidate genes, and also the mutations that appeared at generation two of GmL07 and then fixed, which yielded nine candidate genes (Fig. 5c).

### Disrupted carbon catabolite repression pathway in mutualistic *E. coli*

**Extended Data Fig. 8:**
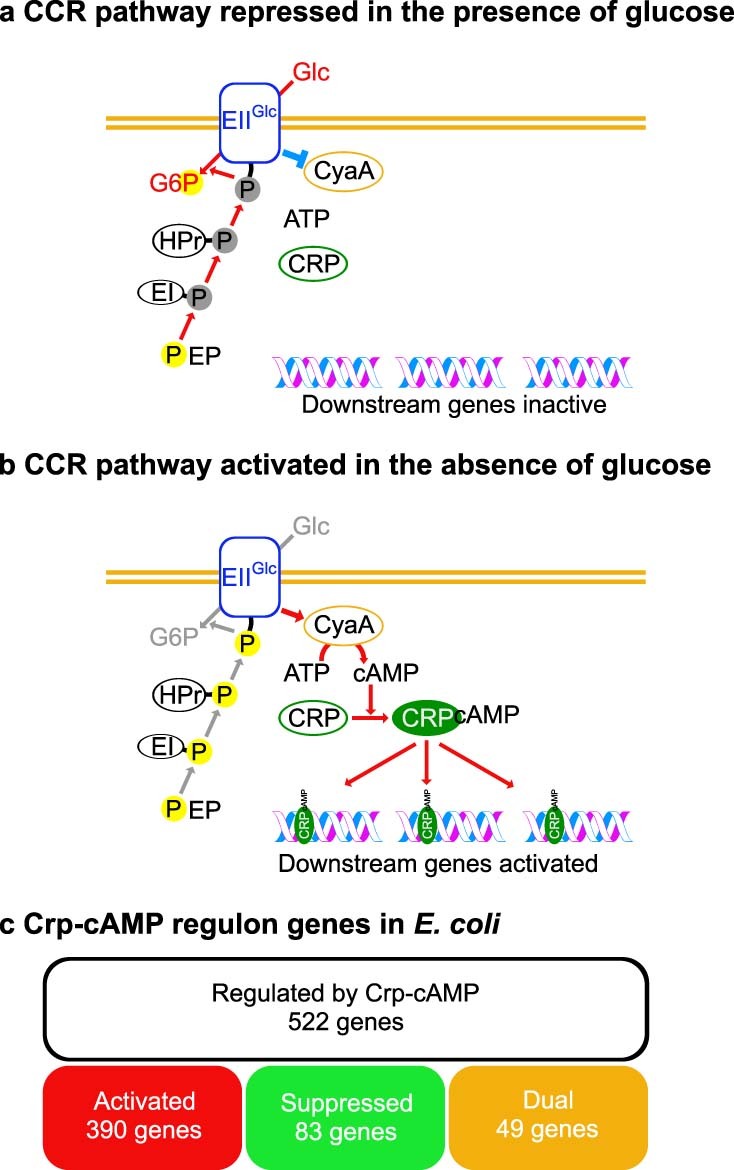
Carbon catabolite repression (CCR) pathway and Crp-cAMP regulon of *E. coli*. **a**, CCR pathway repressed in the presence of glucose. **b**, CCR pathway activated in the absence of glucose. **c**, Number of genes constituting the Crp-cAMP regulon of *E. coli* estimated by RegulonDB^22^. Source data

Of these candidates, we focused on a frameshift mutation that disrupted adenylate cyclase (CyaA) in CmL05 and a non-synonymous mutation that changed a functionally important cAMP binding site of the cAMP receptor protein (Crp) from leucine to proline in GmL07 (Fig. 5d). Despite their independent origins in distinct evolutionary lines, CyaA and Crp are pivotal components of the same global metabolic regulatory system, the carbon catabolite repression (CCR) pathway, operating in diverse bacteria including *E. coli*^18,19^ (Fig. 5e). With sufficient availability of glucose as the primary carbon source for *E. coli*, the CCR components are subjected to glucose-mediated suppression, being in an unphosphorylated form incapable of activating CyaA, by which the intracellular cAMP is maintained at a low level (Extended Data Fig. 8a). When glucose is used up, the glucose-mediated suppression is released, by which the CCR components are phosphorylated and activate CyaA, which results in an elevated intracellular cAMP level and promotes allosteric binding of cAMP to Crp. The resultant global transcriptional regulator Crp-cAMP activates and/or represses several hundreds of operons throughout the bacterial genome, referred to as the Crp-cAMP regulon, by which the bacterial metabolic pathways are switched to exploit other carbon sources for adaptation to nutrient-deficient and/or high bacterial density conditions (Extended Data Fig. 8b)^20,21^. According to RegulonDB^22^, the Crp-cAMP regulon of *E. coli* consists of some 390 upregulated genes and 80 downregulated genes (Extended Data Fig. 8c), which are involved in, for example, upregulation of transporters and catabolic enzymes for non-glucose sugars^23^, quorum sensing induction^24^ and production of extracellular matrix^25^.

**Extended Data Fig. 9:**
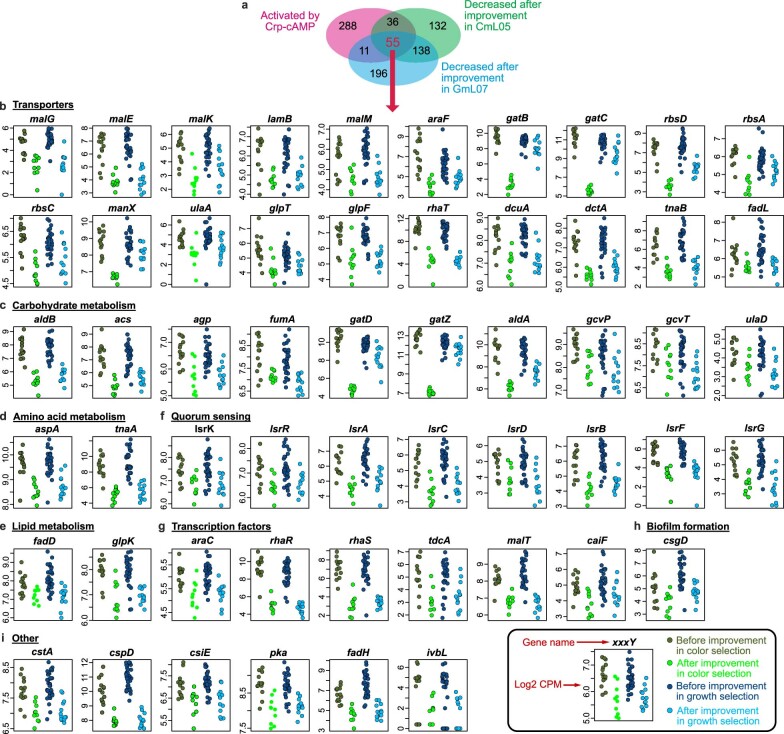
Genes commonly down-regulated in GmL07 and CmL05 after the improvement of host phenotypes, and also down-regulated by disruption of Crp-cAMP in *E. coli*. **a**, Venn diagram showing the commonly down-regulated genes. **b**–**i**, Expression levels of the commonly down-regulated genes in GmL07 and CmL05 after the improvement of host phenotypes. (**b**) Transporter genes. (**c**) Carbohydrate metabolism genes. (**d**) Amino acid metabolism genes. (**e**) Lipid metabolism genes. (**f**) Quorum sensing genes. (**g**) Transcription factor genes. (**h**) Biofilm (= Curli fimbriae) formation genes. (**i**) Other genes. The inset figure at the bottom right represents the explanations of the elements in the plots. The numbers of the biological replicates and exact FDR q-values are provided with the source data. Source data

Both the *cyaA* mutation in CmL05 and the *crp* mutation in GmL07 are disruptive of the CCR pathway. Considering that *E. coli* cells are packed in the host symbiotic organ very densely (Fig. 1k and Extended Data Fig. 2i,k), it seems likely that the symbiotic *E. coli* may be under a nutrient-limited condition in the host insect, at least locally. If so, it is expected that, in the evolutionary *E. coli* lines, while the Crp-cAMP transcriptional regulator was activated before the mutations occurred, the activation was disabled after the mutations occurred. Notably, of 193 genes commonly downregulated after the *yaA* mutation in CmL05 and the *crp* mutation in GmL07, 55 genes were reported as being activated by Crp-cAMP (Extended Data Fig. 9a). These genes, which are expected to be silenced on disruption of the CCR system, were significantly downregulated in CmL05 and GmL07, which represented many transporter genes for non-glucose sugars, carbohydrate metabolism genes, quorum sensing genes, extracellular matrix production genes, transcription factor genes and others (Extended Data Fig. 9b–i).

### Disrupted CCR genes make *E. coli* an insect mutualist

**Fig. 6:**
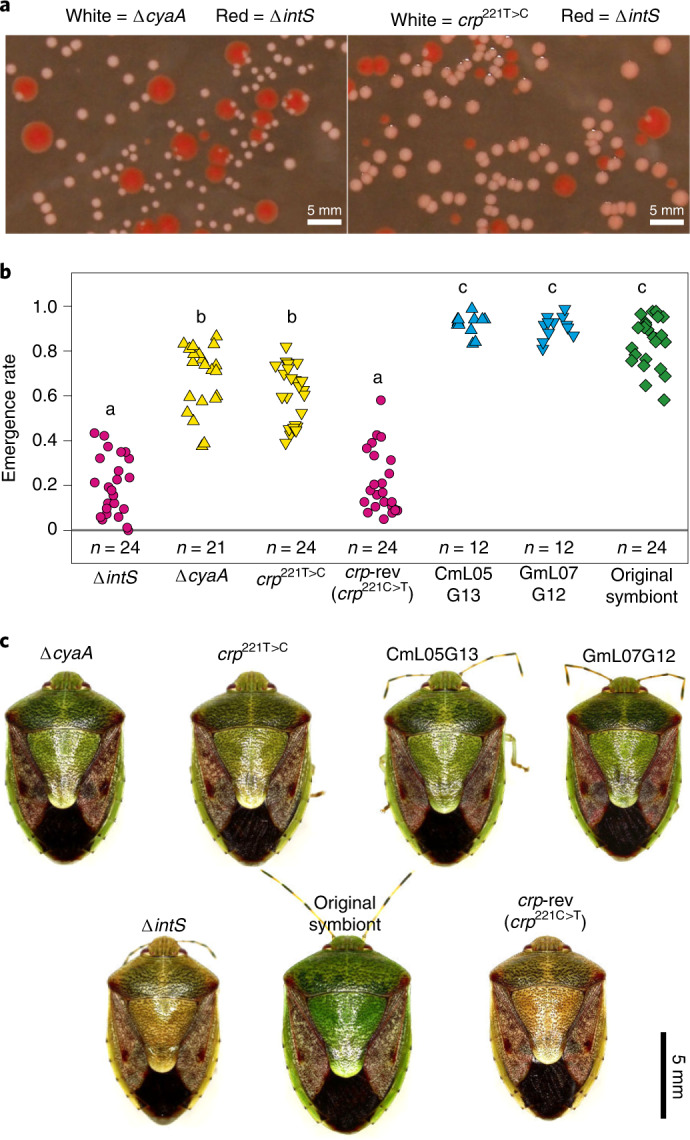
Single mutations disrupting CCR make *E. coli* mutualistic to *P. stali*. **a**, Small, convex and white colonies of Δ*cyaA* and *crp*^221T>C^. **b**, Adult emergence rates of *P. stali* infected with Δ*cyaA* and *crp*^221T>C^. The numbers of biological replicates are shown in the figure. Different alphabetical letters indicate statistically significant differences (two-sided pairwise Wilcoxon rank-sum test with Bonferroni correction: *P* < 0.05). The exact *P* values are provided with the source data. **c**, Adult insects infected with Δ*cyaA* and *crp*^221T>C^, which are larger in size and green in colour in comparison with those infected with control Δ*intS*. Note that the revertant of *crp*^221T>C^, *crp*-rev, exhibits Δ*intS*-like inferior phenotypes. Source data

**Extended Data Fig. 10:**
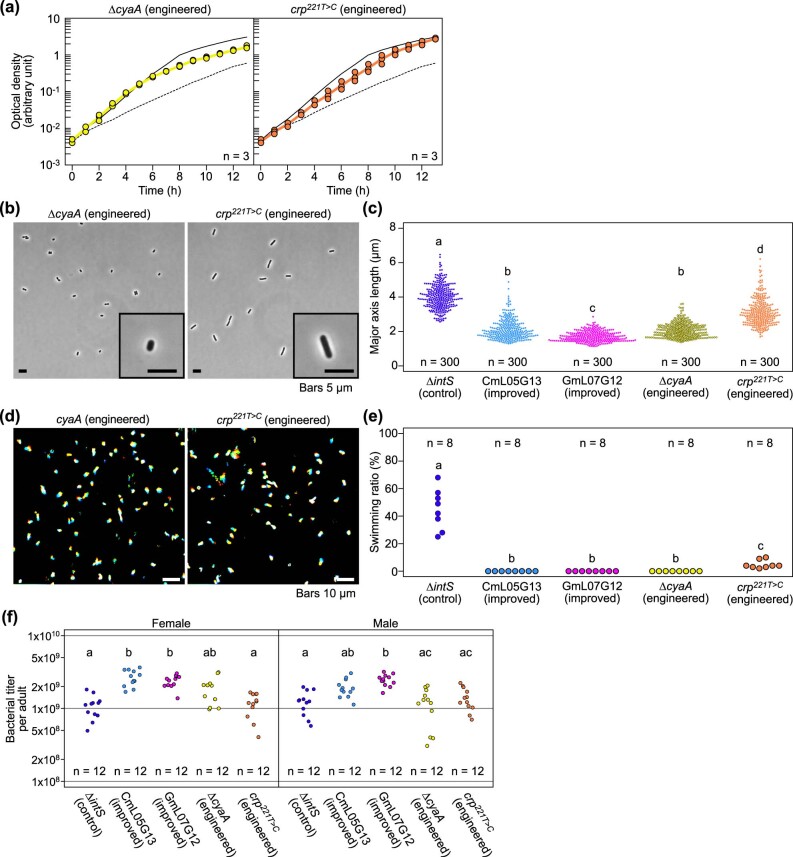
Phenotypic traits of ∆cyaA and crp^221T>C^ mutants of *E. coli*. **a**, Growth curves (3 replicates each). Upper solid line is the trace of Δ*intS* growth curve, whereas lower dotted line is the trace of CmL05 growth curve. **b**, Morphology of bacterial cells. **c**, Quantification of cell size in terms of major axis length. **d**, Motility of bacterial cells visualized by rainbow plot for 2 sec. **e**, Quantification of bacterial motility in terms of number of swimming cells per 100 cells observed. **f**, Bacterial titres in adult females 35 days after emergence in terms of *ntpII* gene copies per insect. In **a**, **c**, **e** and **f**, the numbers of biological replicates are shown in the plots. In **c**, **e** and **f**, different alphabetical letters indicate statistically significant differences (two-sided pairwise Wilcoxon rank sum test with Bonferroni correction: *P* < 0.05). The exact *P*-values are provided with the source data. Source data

To test whether these mutations are involved in the mutualistic traits of the evolutionary *E. coli* lines, we prepared *E.coli* strains that carry the mutations under the wild-type (WT) genetic background: the strain Δ*cyaA* in which the *cyaA* gene is disrupted; and the strain *crp*^221T>C^ whose *crp* gene was engineered to carry the leucine-proline replacement at the cAMP binding site. Both mutant *E. coli* strains exhibited small and convex colonies with little extracellular matrix, somewhat slower growth rate, smaller cell size and loss of flagellar motility (Fig. 6a and Extended Data Fig. 10a–e), which were generally reminiscent of the characteristic traits of the improved evolutionary *E. coli* lines CmL05 and GmL07 (Fig. 3c and Fig. 4a–e). When the mutant *E. coli* strains were inoculated to sterilized newborn nymphs of *P. stali*, both the Δ*cyaA*- and *crp*^221T>C^-infected insects exhibited remarkably high adult emergence rates, which were comparable to the insects infected with the improved evolutionary *E. coli* lines and were significantly higher than the insects infected with the control *E. coli* strains (Fig. 6b). Moreover, the Δ*cyaA*- and *crp*^221T>C^-infected insects were greenish in colour, which were comparable to the greenish insects infected with the improved evolutionary *E. coli* lines and distinct from the dwarf brown insects infected with the control *E. coli* strains (Fig. 6c). On the other hand, the infection densities of *crp*^221T>C^ and Δ*cyaA* were not comparable to those of the improved evolutionary *E. coli* lines (Extended Data Fig. 10f). These results demonstrated that, strikingly, the single mutations that disrupt the CCR global regulator system make *E. coli* mutualistic to the host insect *P. stali*.

## Discussion

We established an experimental insect-*E. coli* symbiotic system in which the model bacterium is localized to the host symbiotic organ, transmissible to the host offspring vertically and supportive of host survival, although not comparable to the original symbiont. By infecting and passaging a hypermutating *E. coli* strain with the host insect trans-generationally, several evolutionary lines rapidly developed improved adult emergence and body colour, realizing recurrent evolution of mutualism in the laboratory. Strikingly, the evolution of *E. coli* into insect mutualist was ascribed to single mutations that convergently disrupted the bacterial CCR pathway, uncovering unexpected involvement of the nutrient-responsive global transcriptional regulator in the establishment of symbiosis.

Our finding sheds light on the evolvability of symbiosis—elaborate mutualistic symbiosis can evolve much more easily and rapidly than conventionally envisaged. We suggest the possibility that the inactivation of the CCR global regulator may represent a pivotal evolutionary step at an early stage of symbiosis. Densely packed in the symbiotic organ, symbiotic bacteria are expected to constantly suffer nutritional shortage and activate the CCR pathway in vain, which may incur substantial metabolic cost and destabilize the symbiotic association. In this context, the disruption of the CCR pathway should benefit and stabilize symbiosis. Our finding may also be relevant to the general evolutionary trend of symbiont genomes towards size reduction^26,27^ and lack of transcription factors^28,29^. The disruption of the CCR pathway causes silencing of otherwise activated about 400 genes under the Crp-cAMP regulon^22^, which accounts for about 10% of the whole *E. coli* genome and provides potential targets for gene disruption, IS amplification and insertion, intragenomic recombination and reductive genome evolution. We propose that, although speculative, inactivation of transcriptional regulators and genome size reduction might have concurrently proceeded in this way during the symbiont genome evolution.

On the other hand, we found that the original symbiont of *P. stali* retained the complete CCR pathway genes, although the uncultivable symbiont accumulated hundreds of pseudogenes in the genome^10^. Plausibly, the CCR disruption observed in the evolutionary *E. coli* lines may represent one of multiple possible evolutionary trajectories towards symbiosis with *P. stali* and we expect that other mechanisms would be uncovered by larger scale and longer-span evolutionary experiments.

The *P. stali*-*E. coli* experimental symbiotic system will open a window to directly observe and analyse the evolutionary processes and mechanisms of mutualistic symbiosis in real time. *E. coli* is among the best understood cellular organisms^30^, whose 4.5–5.5 megabase genome encodes over 4,000 genes and around 70% of them carry functional information^31,32^. Laboratory evolution of mutualism using such a model bacterium with ample technological and genetic resources will lead to understanding of previously unapproachable evolutionary aspects of symbiosis. Considering that *E. coli* represents a universal component of the gut microbiome of human, mouse and other vertebrates^33,34^, the insect-*E. coli* system in combination with the germ-free mouse-*E. coli* experimental evolution systems^35,36^ would enable us to pursue not only the differences but also the commonality underpinning the mechanisms of gut symbiosis across vertebrates and invertebrates.

## Methods

### Insect and bacterial strains used in this study

An inbred laboratory strain of the brown-winged green stinkbug *P. stali* was established from several adult insects collected at Tsukuba, Ibaraki, Japan in September 2012 and has been maintained in the laboratory for years. This strain is associated with an essential and uncultivable gut symbiont *Pantoea* sp. A^10^ in the posterior midgut region specialized as the symbiotic organ (Fig. 1 and Extended Data Fig. 2). The insects were reared on raw peanuts, soya beans and water containing 0.05% ascorbic acid (Merck) at 25 ± 1 °C and 50 ± 5% relative humidity under a long-day regime of 16 h light and 8 h dark. The *E. coli* strains and mutants used in this study are listed in Supplementary Table 4. The mutants ∆*intS*, ∆*mutS* and *crp*^221T>C^ were generated as described later.

### Construction of *E. coli* mutants

The *E. coli* mutant ∆*intS* was established by replacing the *intS* gene of *E. coli* BW25113 with the *nptII* gene that confers kanamycin resistance by λ-Red homologous recombination using the pRed/ET plasmid (Gene Bridges GmbH). The *E. coli* mutant ∆*mutS* was established by replacing the *mutS* gene of ∆*intS* with the FRT-Cm-FRT cassette (Gene Bridges GmbH) by λ-Red homologous recombination; then Cm^R^ was eliminated by Flp-FRT recombination. The *E. coli* mutant *crp*^221T>C^ was established from ∆*intS* by replacing the 221st nucleotide T of the WT *crp* gene with C, which changed the 74th amino acid leucine of the Crp protein to proline. This replacement was introduced by the MAGE method^37,38^ with a 90-mer DNA oligonucleotide (5′-taaagaaatg-atcctctcct-atctgaatca-gggtgatttt-attggcgaac-**C**gggcctgtt-tgaagagggc-caggaacgta-gcgcatgggt-3′) whose 1st to 4th nucleotides were phosphorothioated. To establish the revertant of *crp*^221T>C^ (*crp*^221C>T^), a 90-mer oligonucleotide (5′-taaagaaatg-atcctctcct-atctgaatca-gggtgatttt-attgg**T**gaac-**T**gggcctgtt-tgaagagggc-caggaacgta-gcgcatgggt-3′) was used. This oligonucleotide was designed not only to revert the 221st nucleotide but also replace the 216th nucleotide C to T to introduce a synonymous mutation, which allowed us to discriminate the resultant revertant clone from the unmodified WT.

### Preparation of symbiont-free nymphs by surface sterilization of eggs

Egg clutches produced by the stock culture of *P. stali* were soaked in 4% formaldehyde for 10 min, rinsed with sterilized water several times and kept in sterilized plastic boxes until use. While this treatment does not affect hatchability and survival of the eggs, newborn nymphs fail to acquire the symbiotic bacteria and become symbiont-free^11^.

### Experimental evolution of *P. stali*-*E. coli* artificial symbiotic system

Evolutionary experiments in this study consisted of, for each evolutionary *P. stali* line, (1) preparation of an inoculum either from the *E. coli* culture of Δ*mutS* or Δ*intS* (only G1) or from an adult female of the previous generation (from G2 and on), (2) oral administration of the inoculum to symbiont-free nymphs, (3) rearing of the nymphs either to their adulthood or death, (4) selection of an adult female for inoculation to the next generation, (5) contamination check of the selected adult female, (6) preparation of an inoculum and a glycerol stock from the symbiotic organ dissected from the selected female and (6) morphological measurements of all adult insects obtained.

Either diluted *E. coli* culture (2.5 ml adjusted to OD_600_ = 0.1) or homogenate of the symbiotic organ dissected from a selected female of the previous generation (2.5 ml containing 1/2 organ equivalent) was soaked in a cotton pad and orally administered to around 84 symbiont-free hatchlings derived from 6 surface-sterilized egg masses, by making use of the nymphal behaviour that, after egg surface probing for about 30 min and resting for around 1 d, they take water without feeding and moult to second instar in a few days^10,11^. These nymphs were reared on sterilized peanuts, soya beans and ascorbic acid water as described previously^11^. In the evolutionary experiments, two selection schemes, growth and colour selection, were conducted (Fig. 2). In the growth selection lines (GmL for hypermutating Δ*mutS* lines; GiL for non-mutating Δ*intS* lines), the first-emerged adult female was subjected to dissection of the symbiotic organ for inoculation to the next generation as well as freeze storing. In the colour selection lines (CmL for the Δ*mutS* lines; CiL for the Δ*intS* lines), adult females were collected for 35 d after hatching or until at least 1 adult female emerged. These adult females were anaesthetized on ice and photographed from the ventral side using a digital camera. Their body colour was measured using the image analysing software Natsumushi v.1.10 (ref. ^39^); the adult female that exhibited the highest hue angle (= greenness) was subjected to dissection of the symbiotic organ for inoculation to the next generation as well as freeze-storing.

The symbiotic organ of the selected female was dissected in PBS (0.8% NaCl, 0.02% KCl, 0.115% Na_2_HPO_4_, 0.02% KH_2_PO_4_, pH 7.4), rinsed with 70% ethanol and homogenized in 200 µl sterile water. Of the 200-µl homogenate, 5 µl was used for contamination checking by quantitative PCR (qPCR). The number of *E. coli* genome copies was evaluated in terms of kanamycin resistance gene copies, which are present in the Δ*intS* and Δ*mutS* mutants but absent in WT *E. coli* and other bacteria. The number of total bacterial genome copies was evaluated based on bacterial 16S ribosomal RNA gene copies. When the former *E. coli* genome copy number was approximately the same as the latter bacterial genome copy number, the specimen was diagnosed as free of contamination. When the specimen was diagnosed as contaminated, the next best female was used. For qPCR, the primers Tn5-1789F (5′-TGCTCGACGTTGTCACTGAA-3′) and Tn5-1879R (5′-GCAGGAGCAAGGTGAGATGA-3′) were used for the kanamycin resistance gene, while the primers 16S-967F (5′-CAACGCGAAGAACCTTACC-3′) and 16S-1046R (5′-CGACAGCCATGCANCACCT-3′) were used for the bacterial 16S rRNA gene. The PCR reaction was performed using the Brilliant PCR Mix (Agilent Technologies). The standard curve was drawn using serially diluted ∆*intS* genomic DNA, which contains one kanamycin gene copy and seven 16S rRNA gene copies per genome. The thermal profile was the initial denaturation at 95 °C for 3 min followed by 40 cycles of incubation at 95 °C for 5 s and at 60 °C for 10 s. To confirm specific amplification, melting curve analysis was also included. The reaction was conducted on Mx3000p (Agilent Technologies). While 100 µl of the homogenate of the female symbiotic organ diagnosed as free of contamination was used as the inoculum to the next generation, the remaining homogenate (approximately 95 µl) was mixed with an equal volume of 20% glycerol and stored at −80 °C.

### Inoculation of *E. coli* frozen stocks to *P. stali*

The frozen glycerol stocks were thawed; 50 μl was taken and diluted with sterile water to 3 ml. Each of 3 replicates of around 84 symbiont-free hatchlings from 6 surface-sterilized egg masses was fed with 1 ml inoculum soaked in a cotton pad as described above. The symbionts A and Δ*mutS* were included in the evaluation as positive and negative controls, respectively. Adult emergence of the insects was monitored for 50 d after hatching. All the adult insects were photographed from the dorsal side with a digital camera; the hue angle (= greenness) of the scutellum and thorax width were measured with ImageJ v.1.53^40^. For the subsequent RNA sequencing (RNA-seq) analyses and resequencing of *E. coli* genomes, the symbiotic organs were isolated from the adult insects and homogenized in 100 µl PBS. Of the 100 µl homogenate, 50 µl was subjected to RNA-seq and the remaining 50 µl was used for genome resequencing.

### RNA-seq analyses

The homogenate of the symbiotic organ was subjected to total RNA extraction using RNAiso (Takara Bio) and the RNeasy Mini Kit (QIAGEN). Then, rRNAs of both insect and bacterial origins were removed from the total RNA samples using the Ribo-Zero Gold rRNA Removal Kit (Epidemiology) (Illumina). The rRNA-depleted RNAs were converted to paired-end libraries using the Sure Select Strand Specific RNA Kit (Agilent Technologies) or TruSeq RNA Library Prep Kit v2 (Illumina) (Supplementary Table 1). The libraries were sequenced with HiSeq 3000 or HiSeq X (Illumina). The obtained sequences were trimmed, mapped to the *E. coli* BW25113 genome sequence (accession no. NZ_CP009273) and read-counted with CLC Genomics Workbench 10.0 (QIAGEN). Normalizations and differential expression analyses were conducted with EdgeR v.3.32.1 (ref. ^41^). Complex Heatmap v.2.10.0 (ref. ^42^) was used for the clustering analyses and to draw the heatmaps of the RNA-seq libraries.

### Genome resequencing and detection of structural changes

DNA samples were extracted from the homogenates of the symbiotic organ using the QIAamp DNA Mini Kit (QIAGEN). The extracted DNAs were converted to paired-end libraries using the Nextera XT DNA Library Prep Kit (Illumina) and the libraries were sequenced with MiSeq system (Illumina). CLC Genomic Workbench v.10.0 was used to detect the *E. coli* genome variants that emerged during the evolutionary experiments. The heatmaps of the variant frequency data were drawn using Complex Heatmap v.2.10.0 (ref. ^42^).

### Fluorescence in situ hybridization

Fluorescence in situ hybridization (FISH) analyses were performed essentially as described by Koga et al.^43^. The whole insect bodies or isolated digestive tracts were fixed with PBS containing 4% formaldehyde (Fujifilm). The fixed samples were embedded in Technovit 8100 (Kulzer) and processed into 2-µm tissue sections using a rotary microtome RM2255 (Leica Biosystems). The Alexa Fluor 555-labelled oligonucleotide probes Eco934 (5′-CATGCTCCACCGCTTGTG-3′) and SymAC89R (5′-GCAAGCTCTTCTGTGCTGCC-3′) were used to detect *E. coli* and symbiont A, respectively^14^. Host nuclei were counterstained with 4′,6-diamidino-2-phenylindole (Dojindo). The hybridized specimens were observed using a fluorescence dissection microscope M165FC with Leica Application Suite v.4.13.0 (Leica Microsystems), an epifluorescence microscope DM6B with Leica Application Suite X v.3.7.1.21655 (Leica Microsystems) and a laser confocal microscope LSM700 with ZEN 2011 v.7.0.7.0 (ZEISS). For Fig. 1d and Extended Data Fig. 2j, panoramic images were constructed by merging multiple images using Affinity Photo v.1.10.5 (Serif Ltd).

### Infection of *E. coli* mutants and effects on host phenotypes

*E. coli* mutants were cultured, diluted and orally administrated to symbiont-free newborn nymphs of *P. stali* as described above. The insects were reared to monitor their adult emergence for 42 d after hatching. The dorsal images of the adults were taken with an image scanner GT-X830 (Epson) and the hue angle of the scutellum and thorax width was measured and analysed using the Natsumushi software v.1.10^39^. *P. stali* harbouring the original symbiont *Pantoea* sp. A was also included as a reference. As for the adult females infected with *E. coli*, bacterial titres in the symbiotic organs were measured by qPCR. The KAPA SYBR FAST qPCR Kit (Roche) and Tn5-1789F and Tn5-1879R primer sets were used for quantification. The standard curves were drawn using serially diluted pT7Blue (Takara Bio) plasmid carrying a kanamycin resistance gene fragment. The qPCR reactions were conducted on the Light Cycler 96 (Roche).

### Measurement of *E. coli* phenotypes

To inspect colony morphology and extracellular matrix production, *E. coli* cultures were spread onto lysogeny broth agar plates containing 80 µg ml^−1^ Congo Red (Merck) and incubated at 25 °C for 3 d. Colonies formed on the plate were photographed by using a scanner GT-X850 and/or dissection microscope S9i (Leica Microsystems).

For growth curve measurements, each glycerol stock of *E. coli* was inoculated to 2 ml lysogeny broth (Becton Dickinson) and incubated at 25 °C for 16 h with shaking at 200 r.p.m. The cell culture was diluted to OD_600_ = 0.005 in 25 ml lysogeny broth and incubated at 25 °C with shaking at 200 r.p.m. From the bacterial culture, 120 µl of cell suspension was sampled every hour and samples were subjected to the measurement of OD_600_ using a spectrometer UV-1800 (Shimadzu).

For time-lapse analyses of growth and morphology of individual *E. coli* cells, two types of microfluidic devices were used. One type was a microfluidic device where bacterial cells were enclosed in microchambers etched on a glass coverslip. A cellulose membrane was attached to a coverslip via biotin-streptavidin binding, on which the microchambers were created as described elsewhere^44,45^. Another type was a microfluidic device made of polydimethylsiloxane with a channel structure similar to Mother Machine as described by Wang et al.^46^ (Fig. 4f). The width of the cell observation channels in this device was 9 µm, which was broader than that of the Mother Machine; thus, each cell observation channel could harbour 30–70 individual *E. coli* cells depending on cell size. *E. coli* cells in the exponential phase were introduced into both types of microfluidic devices and observed under a Nikon Ti-E microscope equipped with an ORCA-fusion camera (Hamamatsu Photonics). In the time-lapse measurements, phase-contrast images were acquired with a 100× oil immersion objective lens (plan apochromat, numerical aperture 1.45) at an interval of 3 min, in which 50–100 XY positions were simultaneously observed. The microscope was controlled from a computer using Micro-Manager v.4. In the microchamber device measurements, lysogeny broth was supplemented with 0.1% bovine serum albumin and 0.02% Tween-80 to suppress cell adhesion and was introduced into the devices at a flow rate of 2 ml h^−1^.

For measurements of size and flagellar motility, *E. coli* cells were grown in lysogeny broth medium with shaking at 25 °C to around OD_600_ = 2.0, observed under a phase-contrast microscope IX71 (Olympus), recorded by a charge-coupled device camera DMK33UP5000.WG (The Imaging Source) at 30 frames per second and analysed using ImageJ v.1.53 (ref. ^40^) and IGOR Pro 8.02J (WaveMetrics). The cell size data were measured for six individual cultures. The swimming ratio data were obtained as the number of swimming cells in 100 cells from 8 individual cultures.

### Statistics and reproducibility

Statistical analyses were conducted with R v.4.1.2 (ref. ^47^) and RStudio^48^. R was also used to plot the data. No statistical methods were used to predetermine sample sizes, but our sample sizes are similar to those reported in previous publications^10,11,14^. All data points were plotted as long as applicable. Data distribution was assumed to be normal but this was not formally tested. For Figs. 1, 4 and 6, and Extended Data Figs. 1 and 10, the numbers of biological replicates are shown in the figures. Exact *P* values are provided with the source data. For Fig. 1a–d,f,h–k,m and Extended Data Figs. 2 and 5, at least two replicate analyses were conducted, and all replicates gave essentially the same results.

### Reporting summary

Further information on research design is available in the Nature Research Reporting Summary linked to this article.

## Supplementary information

Supplementary Information Supplementary Tables 1–4.

Reporting Summary

Supplementary Video 1 Newborn nymphs of *P. stali* probing the eggshell to acquire the symbiotic bacteria.

Supplementary Video 2 Slow growth of the evolutionary *E. coli* lines GmL07 and CmL05 at the single-cell level.

Supplementary Video 3 Motility of the WT *E. coli* strain BW25113, the control *E. coli* strains Δ*ιntS* and Δ*mutS* and the improved evolutionary *E. coli* lines GmL07 and CmL05.

Supplementary Video 4 Characteristic cell shapes and growth modes of *E. coli* cells in microfluidic channels.

## Data Availability

All RNA and DNA sequencing data produced in this study were deposited in the DNA Data Bank of Japan (DDBJ) Sequence Read Archive (Supplementary Tables 1 and 2). The data have been deposited with links to BioProject accession no. PRJDB5544 in the DDBJ BioProject database. Source data are provided with this paper.

## Source data

Source Data Fig. 1 Numeric source data for Fig. 1e,l.
Source Data Fig. 3 Numeric source data for Fig. 3a,b,d,e.
Source Data Fig. 4 Numeric source data for Fig. 4a,c,e,g,h and statistical source data for Fig. 4.
Source Data Fig. 5 Numeric source data for Fig. 5a,b,c.
Source Data Fig. 6 Numeric source data for Fig. 6b and statistical source data for Fig. 6.
Source Data Extended Data Fig. 1 Numeric source data for Extended Data Fig. 1a,b,c.
Source Data Extended Data Fig. 3 Numeric source data for Extended Data Fig. 3a,b.
Source Data Extended Data Fig. 4 Numeric source data for Extended Data Fig. 4a,b,c,d,e,f,g,h.
Source Data Extended Data Fig. 6 Numeric source data for Extended Data Fig. 6a,b,c and statistical source data for Extended Data Fig. 6.
Source Data Extended Data Fig. 7 Numeric source data for Extended Data Fig. 7.
Source Data Extended Data Fig. 8 Numeric source data for Extended Data Fig. 8c.
Source Data Extended Data Fig. 9 Numeric source data for Extended Data Fig. 9a–i and statistical source data for Extended Data Fig. 9.
Source Data Extended Data Fig. 10 Numeric source data for Extended Data Fig. 10a,c,e,f and statistical source data for Extended Data Fig. 10.
