# Supplementary material for: Single mutation makes Escherichia coli an insect mutualist
Source: Nat Microbiol. 2022 Aug 4;7(8):1141–50. doi: 10.1038/s41564-022-01179-9 (PMC9352592; doi:10.1038/s41564-022-01179-9)
Supplement: Supplementary file 2 — Reporting Summary [file 41564_2022_1179_MOESM2_ESM.pdf]

nature portfolio

Corresponding author(s): Takema Fukatsu

Last updated by author(s): Jun 6, 2022

Reporting Summary

Nature Portfolio wishes to improve the reproducibility of the work that we publish. This form provides structure for consistency and transparency in reporting. For further information on Nature Portfolio policies, see our Editorial Policies and the Editorial Policy Checklist.

Statistics

For all statistical analyses, confirm that the following items are present in the figure legend, table legend, main text, or Methods section.

n/a	Confirmed	
☐	☒	The exact sample size (*n*) for each experimental group/condition, given as a discrete number and unit of measurement
☐	☒	A statement on whether measurements were taken from distinct samples or whether the same sample was measured repeatedly
☐	☒	The statistical test(s) used AND whether they are one- or two-sided<br>*Only common tests should be described solely by name; describe more complex techniques in the Methods section.*
☒	☐	A description of all covariates tested
☐	☒	A description of any assumptions or corrections, such as tests of normality and adjustment for multiple comparisons
☐	☒	A full description of the statistical parameters including central tendency (e.g. means) or other basic estimates (e.g. regression coefficient) AND variation (e.g. standard deviation) or associated estimates of uncertainty (e.g. confidence intervals)
☐	☒	For null hypothesis testing, the test statistic (e.g. *F*, *t*, *r*) with confidence intervals, effect sizes, degrees of freedom and *P* value noted<br>*Give P values as exact values whenever suitable.*
☒	☐	For Bayesian analysis, information on the choice of priors and Markov chain Monte Carlo settings
☒	☐	For hierarchical and complex designs, identification of the appropriate level for tests and full reporting of outcomes
☒	☐	Estimates of effect sizes (e.g. Cohen's *d*, Pearson's *r*), indicating how they were calculated

Our web collection on statistics for biologists contains articles on many of the points above.

Software and code

Policy information about availability of computer code

Data collection	LAS Ver. 4.13.0, LAS X Ver. 3.7.1.21655, ZEN 2011 Ver. 7.0.7.0
Data analysis	CLC Genomics Workbench ver. 10.0, EdgeR ver. 3.32.1, Complex Heatmap ver. 2.10.0, Natsumushi ver. 1.10, Micromanager 4, ImageJ ver. 1.53, IGOR Pro 8.02J, R ver. 4.1.2, RStudio 1.4.1103, Affinity Photo Ver. 1.10.5

For manuscripts utilizing custom algorithms or software that are central to the research but not yet described in published literature, software must be made available to editors and reviewers. We strongly encourage code deposition in a community repository (e.g. GitHub). See the Nature Portfolio guidelines for submitting code & software for further information.

Data

Policy information about availability of data

All manuscripts must include a data availability statement. This statement should provide the following information, where applicable:

- Accession codes, unique identifiers, or web links for publicly available datasets
- A description of any restrictions on data availability
- For clinical datasets or third party data, please ensure that the statement adheres to our policy

The RNAseq and DNAseq reads generated and analyzed in this study are available in the DDBJ (DRA run acc nos. DRR088471--DRR088509, DRR088509 and DRR330401-DRR330422). All other source data and statistical data are provided with the paper.

Field-specific reporting

Please select the one below that is the best fit for your research. If you are not sure, read the appropriate sections before making your selection.

☒ Life sciences ☐ Behavioural & social sciences ☐ Ecological, evolutionary & environmental sciences

For a reference copy of the document with all sections, see nature.com/documents/nr-reporting-summary-flat.pdf

Life sciences study design

All studies must disclose on these points even when the disclosure is negative.

Sample size	Sample sizes were determined as sufficiently large as possible to enable robust statistical tests.
Data exclusions	No data were excluded.
Replication	At least two replicates (generally over three replicates) for each of the analyses were conducted and all replicates produced essentially the same results.
Randomization	All insect materials used in this study were randomly chosen from the stock culture and provided by the technical staffs who were not informed how the materials they provided to the investigators would be used in the experiments conducted by the investigators.
Blinding	The investigators were not involved in the above-mentioned, material providing processes. The investigators only told the technical staffs who maintain the insect stock culture to provide the number of insect materials they needed to conduct the experiments. The experimental groups were prepared from these randomly chosen insects by the technical staffs. The inoculum of the infection experiments were cultured from single colonies selected randomly, with similar size and morphology

Reporting for specific materials, systems and methods

We require information from authors about some types of materials, experimental systems and methods used in many studies. Here, indicate whether each material, system or method listed is relevant to your study. If you are not sure if a list item applies to your research, read the appropriate section before selecting a response.

Materials & experimental systems

n/a	Involved in the study	
☒	☐	Antibodies
☒	☐	Eukaryotic cell lines
☒	☐	Palaeontology and archaeology
☐	☒	Animals and other organisms
☒	☐	Human research participants
☒	☐	Clinical data
☒	☐	Dual use research of concern

Methods

n/a	Involved in the study	
☒	☐	ChIP-seq
☒	☐	Flow cytometry
☒	☐	MRI-based neuroimaging

Animals and other organisms

Policy information about studies involving animals; ARRIVE guidelines recommended for reporting animal research

Laboratory animals	An inbred laboratory strain of the brown-winged green stinkbug Plautia stali, which was established from several adult insects collected at Tsukuba, Ibaraki, Japan in September 2012, and has been maintained in the laboratory for years, was used. The insects were reared on raw peanuts, soybeans and water containing 0.05% ascorbic acid at 25 ± 1˚C and 50 ± 5% relative humidity under a long-day regime of 16 h light and 8 h dark. In the infection experiments randomly chosen eggs were used and thus their sex didn't be specified at the start of the experiments. For qPCR analyses the second instar nymphs whose sex cannot be identified by appearance were used. For the histological analysis, 35-day-old adult females and males were used.
Wild animals	This study did not involve wild animals.
Field-collected samples	This study did not involve samples collected from the field.
Ethics oversight	No ethical approval or guidance was required because this study involved only the stinkbug.

Note that full information on the approval of the study protocol must also be provided in the manuscript.
